# Supplementary figures and images for: KDM5 demethylases suppress R-loop-mediated ‘viral mimicry’ and DNA damage in breast cancer cells
Source: eLife. 2025 Oct 13;14:RP106249. doi: 10.7554/eLife.106249 (PMC12517688; doi:10.7554/eLife.106249)

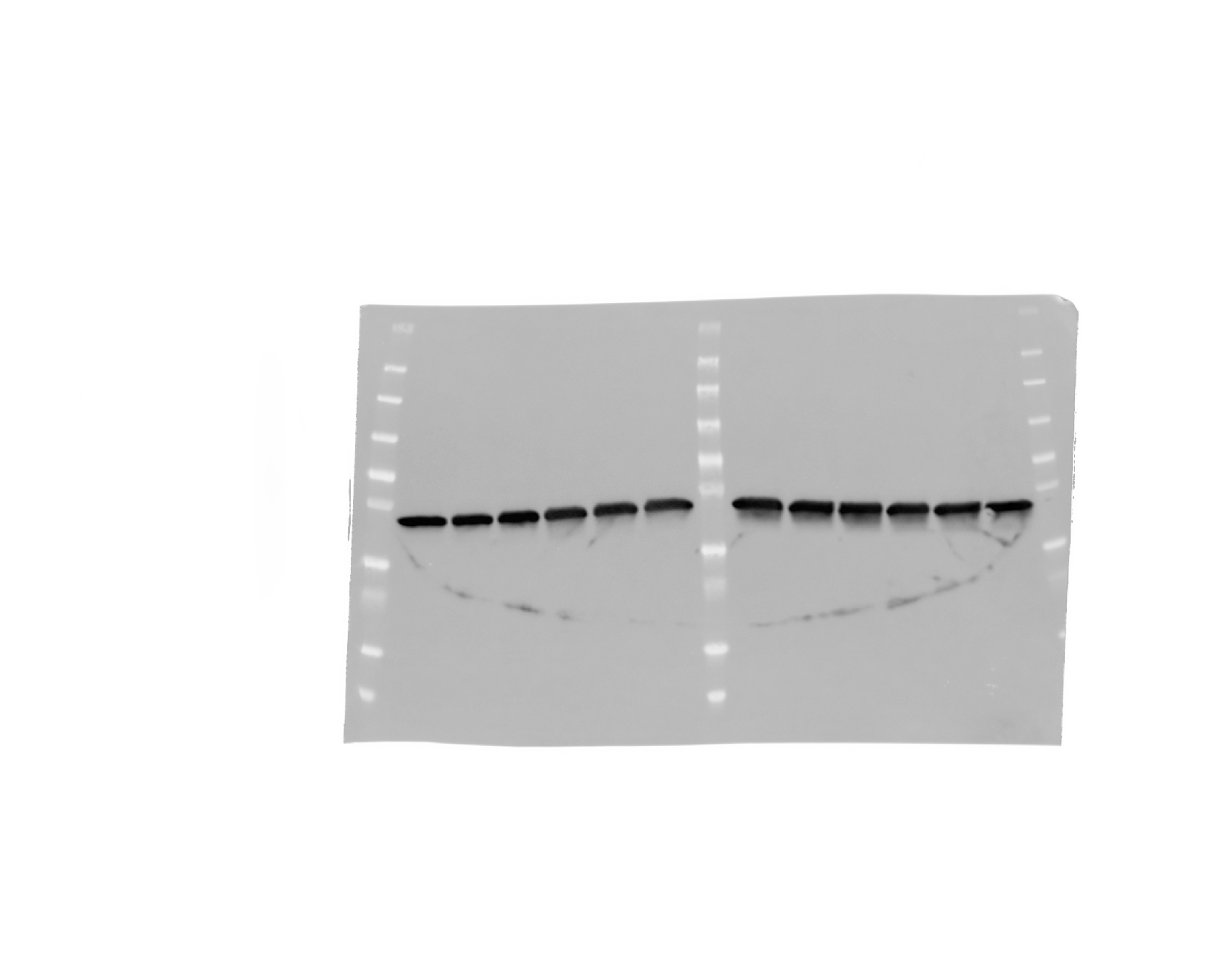

Supplement: Figure 2—figure supplement 1—source data 1. [file elife-106249-fig2-figsupp1-data1.zip › Figure 2-figure supplement 1-Source data 1_unlabelled blots/Figure 2-figure supplement 1b_Actin_unlabelled.tif]

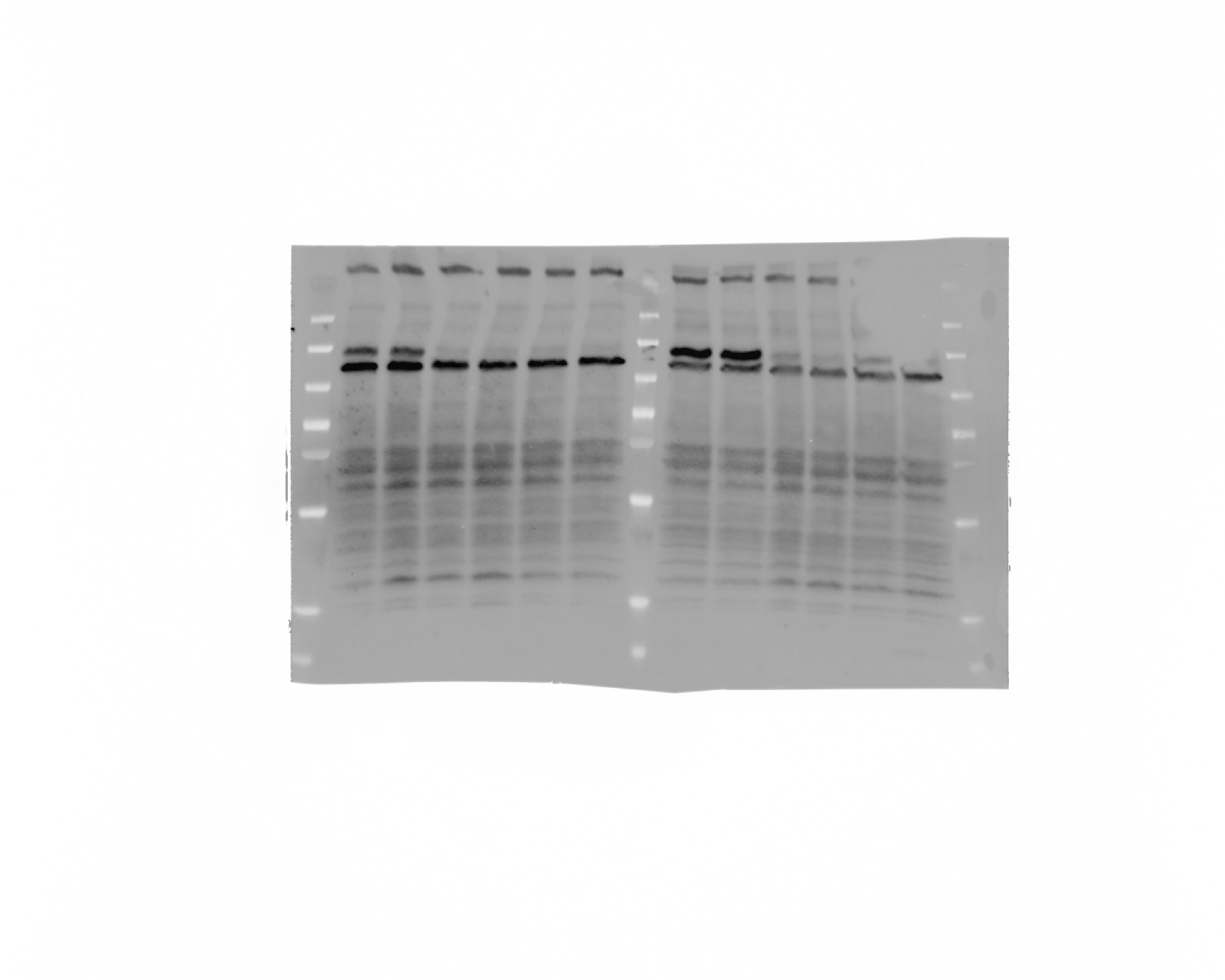

Supplement: Figure 2—figure supplement 1—source data 1. [file elife-106249-fig2-figsupp1-data1.zip › Figure 2-figure supplement 1-Source data 1_unlabelled blots/Figure 2-figure supplement 1b_ADAR_unlabelled.tif]

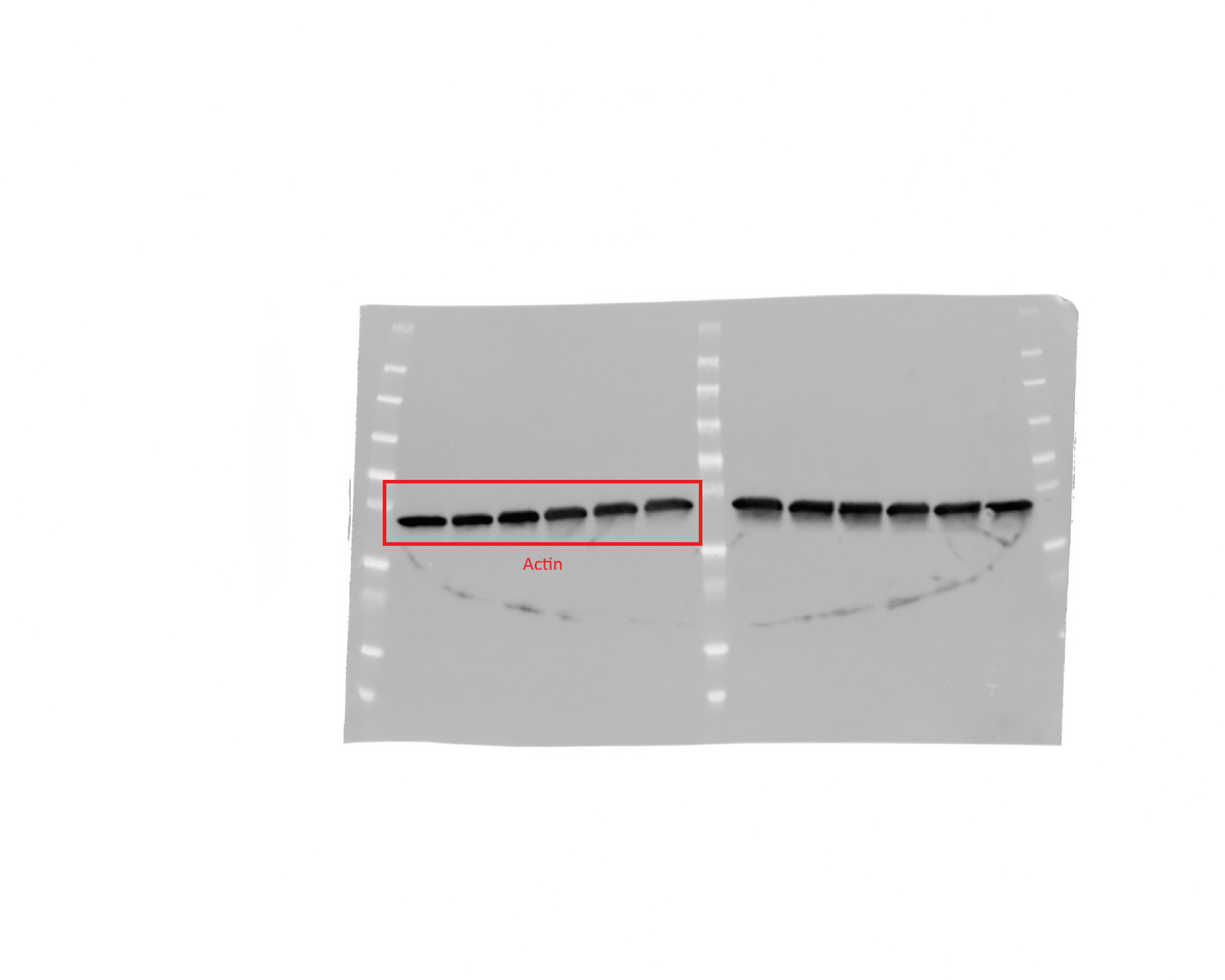

Supplement: Figure 2—figure supplement 1—source data 2. [file elife-106249-fig2-figsupp1-data2.zip › Figure 2-figure supplement 1-Source data 1_labelled blots/Figure 2-figure supplement 1b_Actin_labelled.tif]

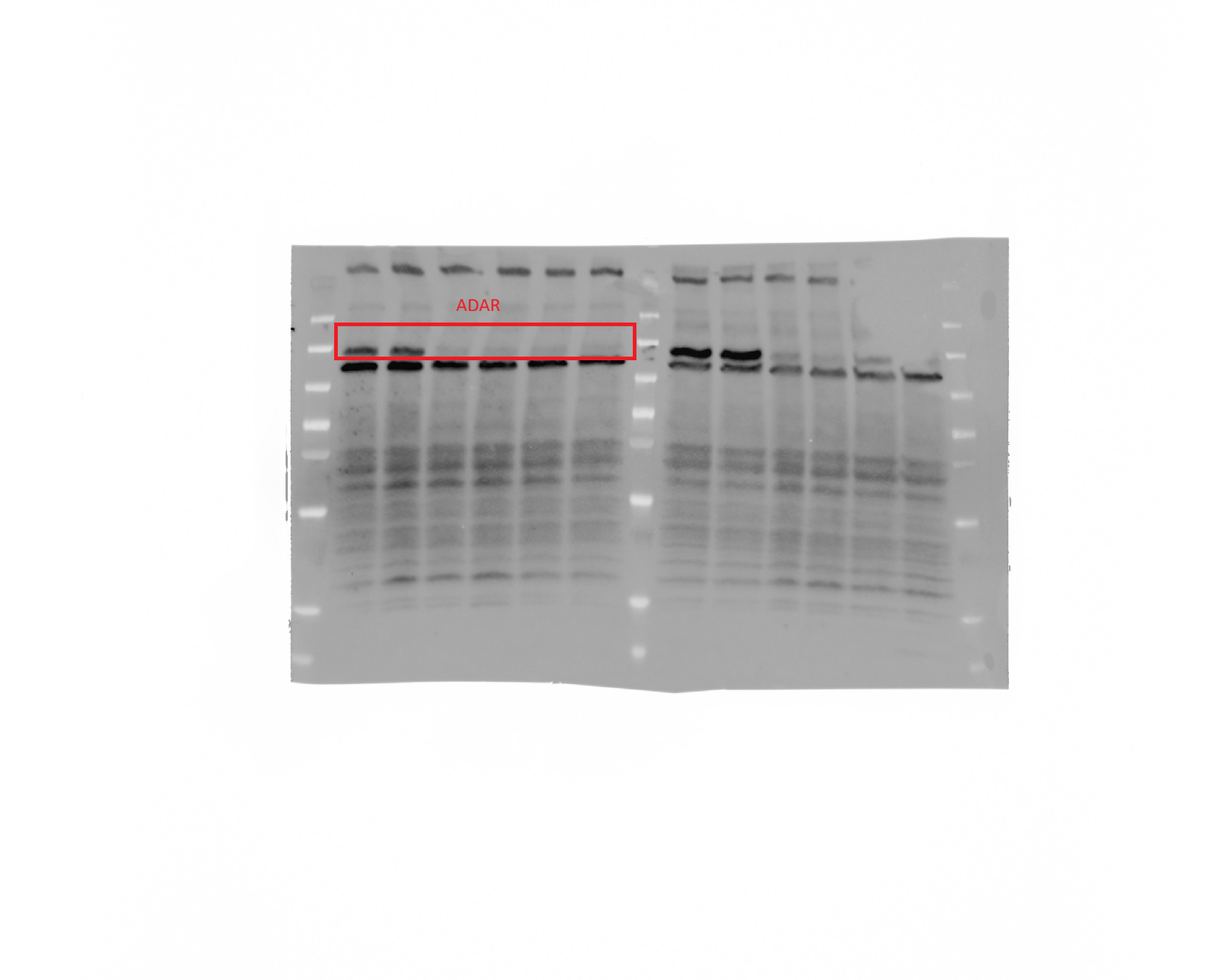

Supplement: Figure 2—figure supplement 1—source data 2. [file elife-106249-fig2-figsupp1-data2.zip › Figure 2-figure supplement 1-Source data 1_labelled blots/Figure 2-figure supplement 1b_ADAR_labelled.tif]

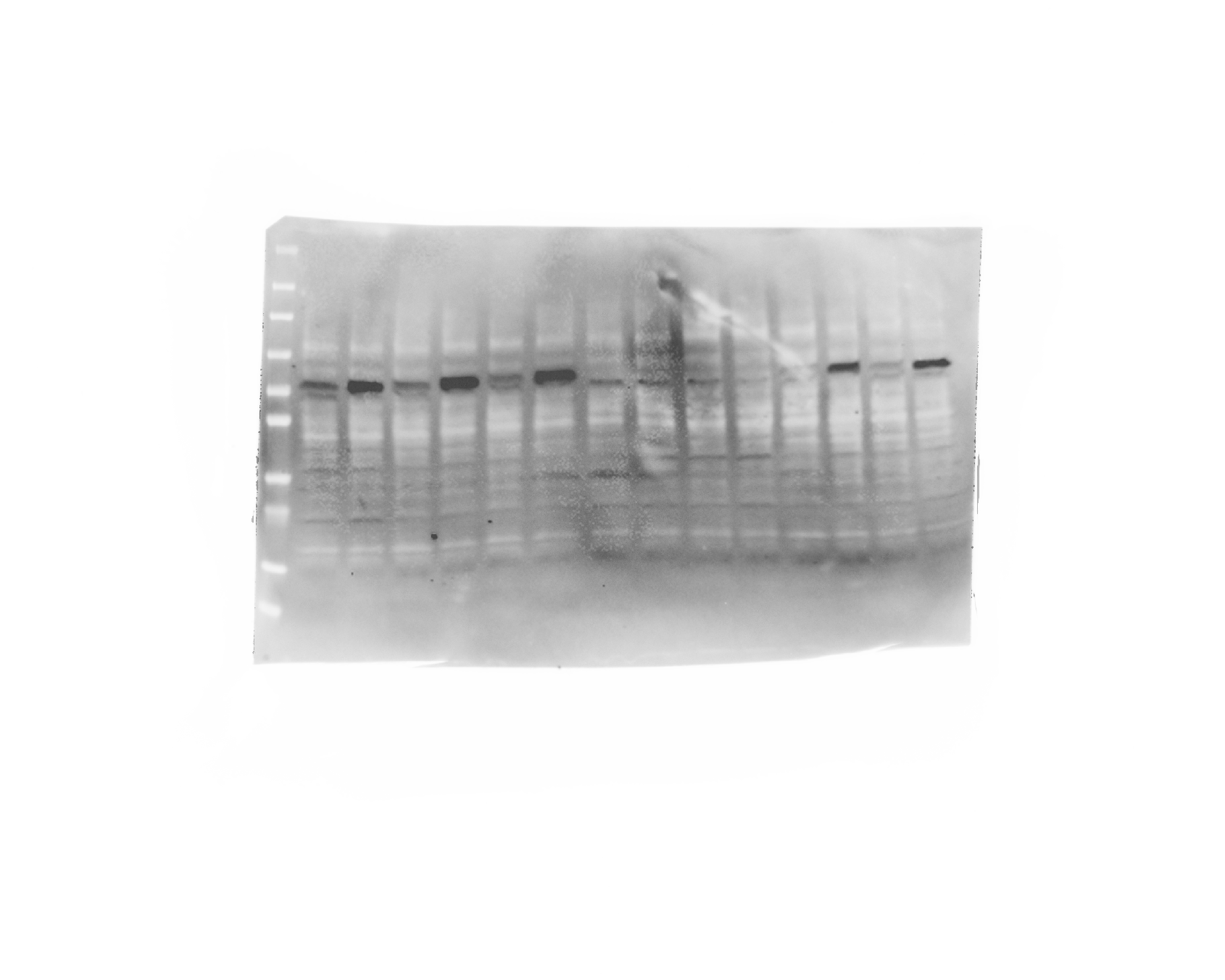

Supplement: Figure 2—figure supplement 1—source data 3. [file elife-106249-fig2-figsupp1-data3.zip › Figure 2-figure supplement 1-Source data 2_unlabelled blots/Figure 2-figure supplement 1c_cGAS_unlabelled.tif]

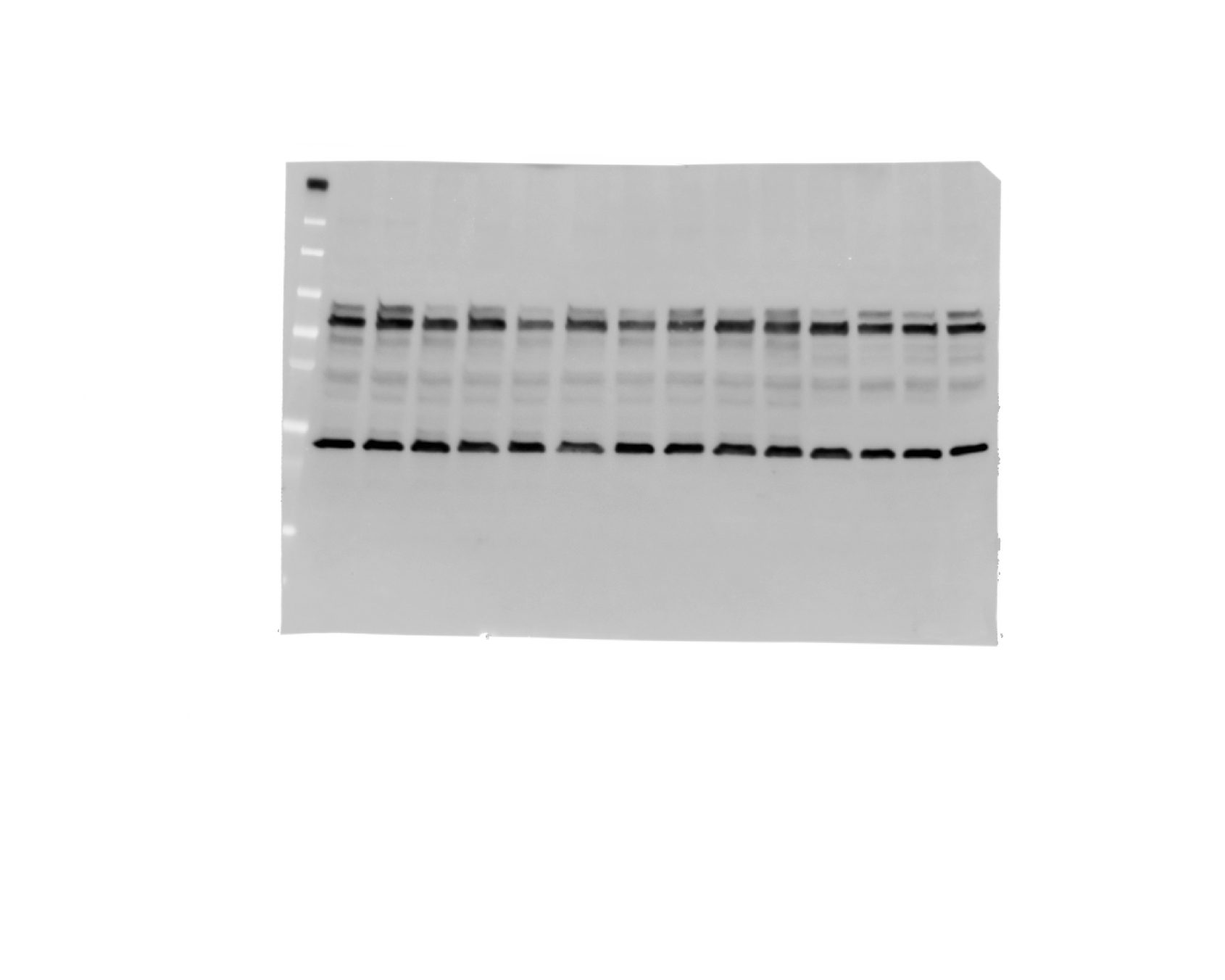

Supplement: Figure 2—figure supplement 1—source data 3. [file elife-106249-fig2-figsupp1-data3.zip › Figure 2-figure supplement 1-Source data 2_unlabelled blots/Figure 2-figure supplement 1c_MAVS_unlabelled.tif]

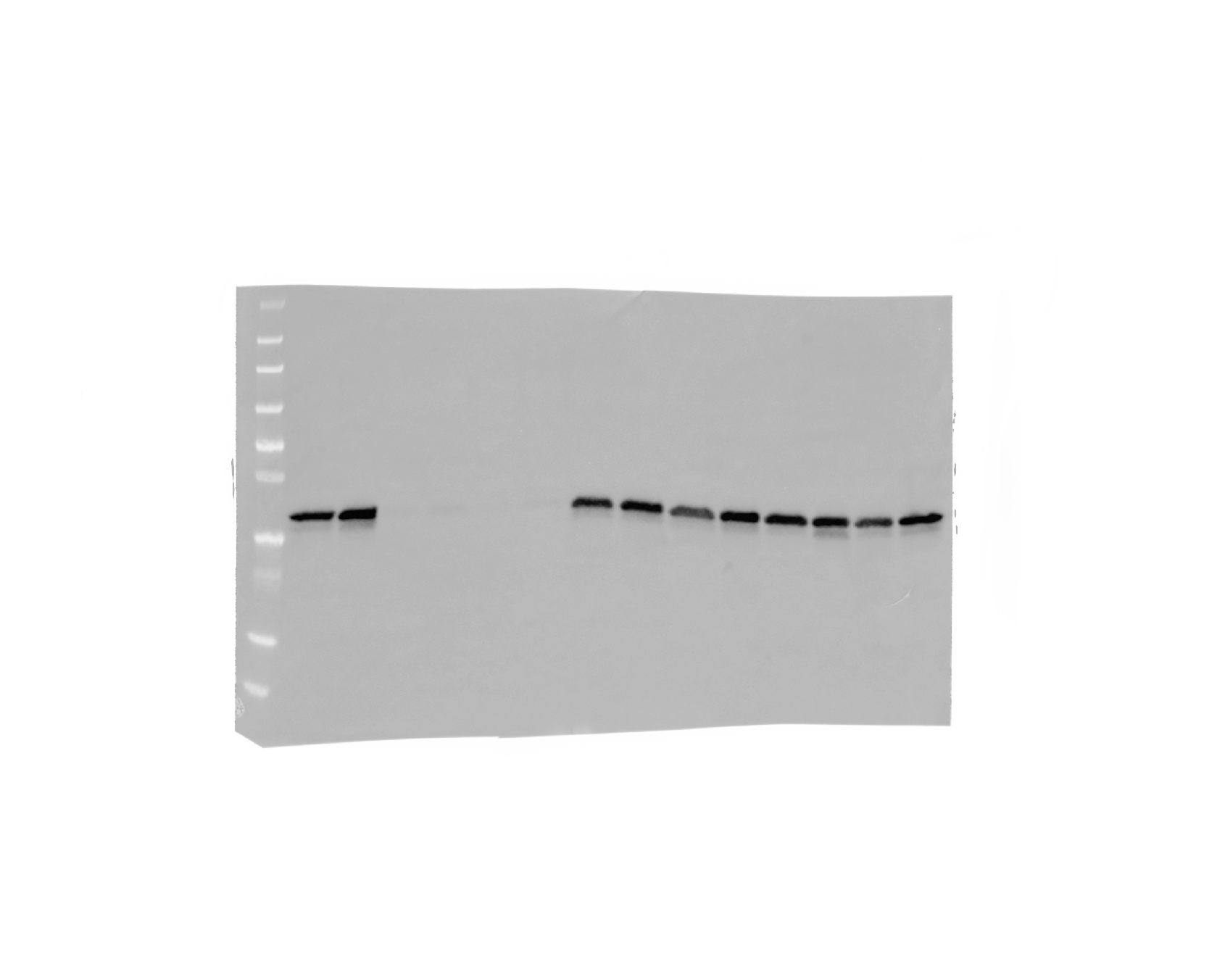

Supplement: Figure 2—figure supplement 1—source data 3. [file elife-106249-fig2-figsupp1-data3.zip › Figure 2-figure supplement 1-Source data 2_unlabelled blots/Figure 2-figure supplement 1c_STING_unlabelled.tif]

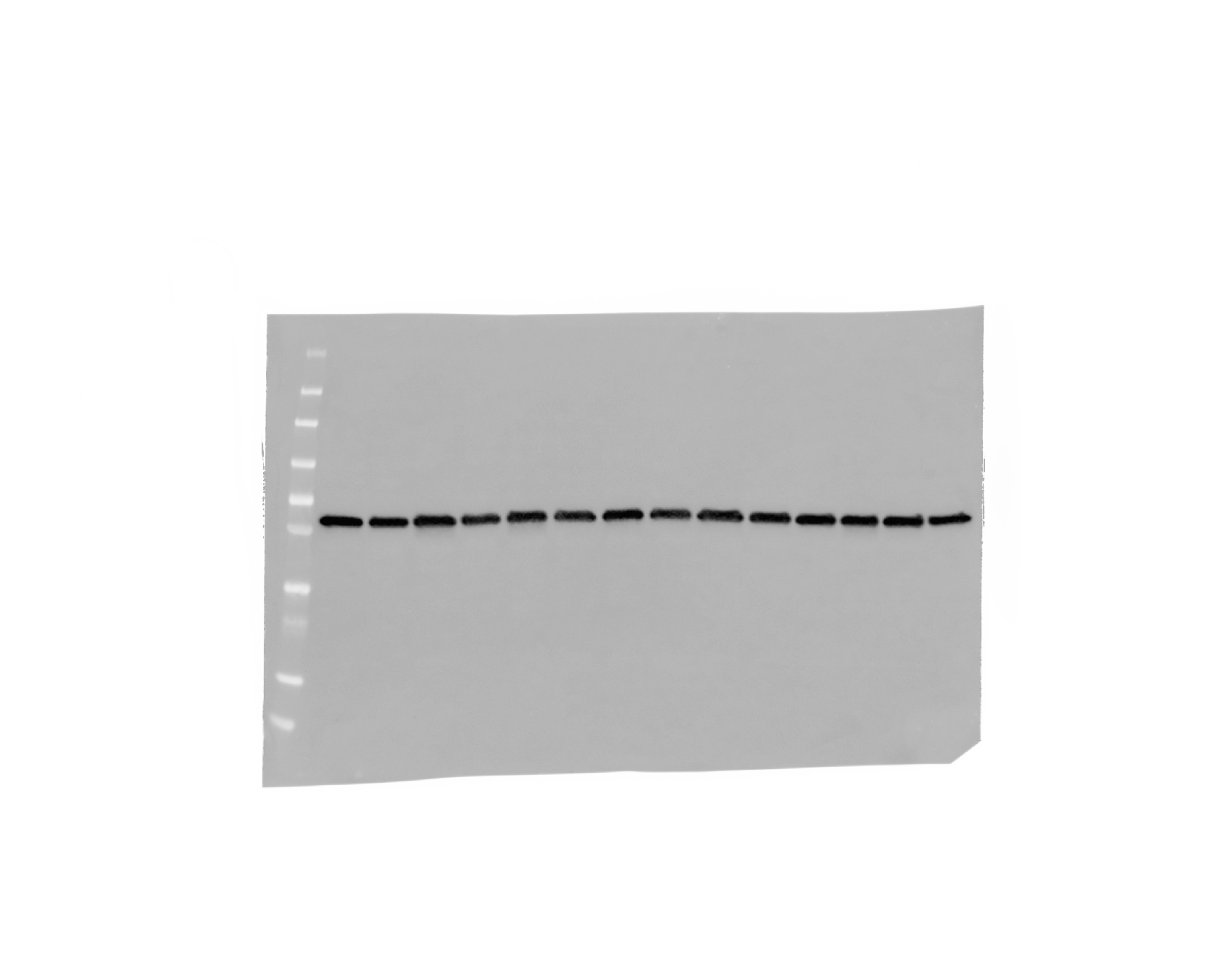

Supplement: Figure 2—figure supplement 1—source data 3. [file elife-106249-fig2-figsupp1-data3.zip › Figure 2-figure supplement 1-Source data 2_unlabelled blots/Figure 2-figure supplement 1c_Tubulin_unlabelled.tif]

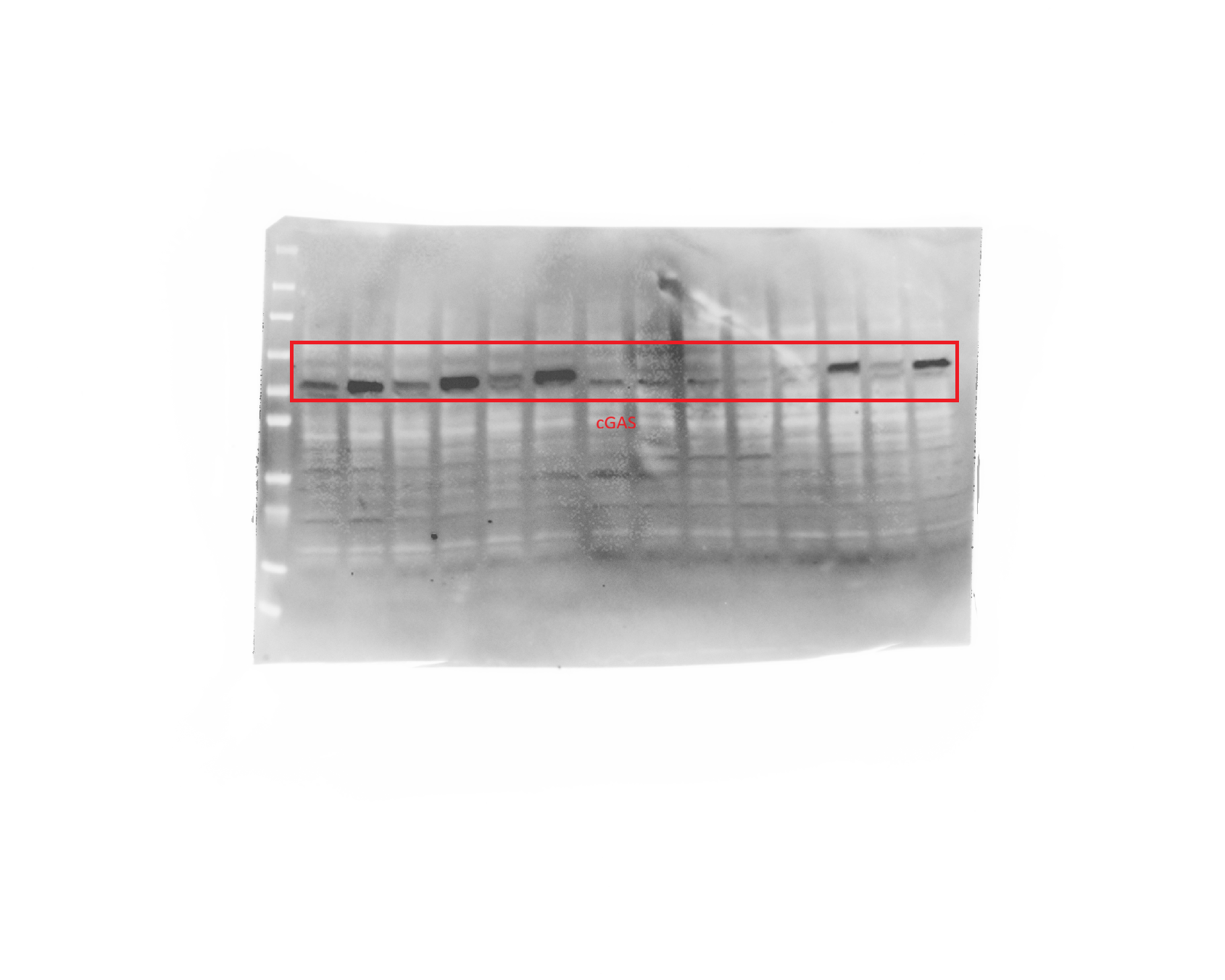

Supplement: Figure 2—figure supplement 1—source data 4. [file elife-106249-fig2-figsupp1-data4.zip › Figure 2-figure supplement 1-Source data 2_labelled blots/Figure 2-figure supplement 1c_cGAS_labelled.tif]

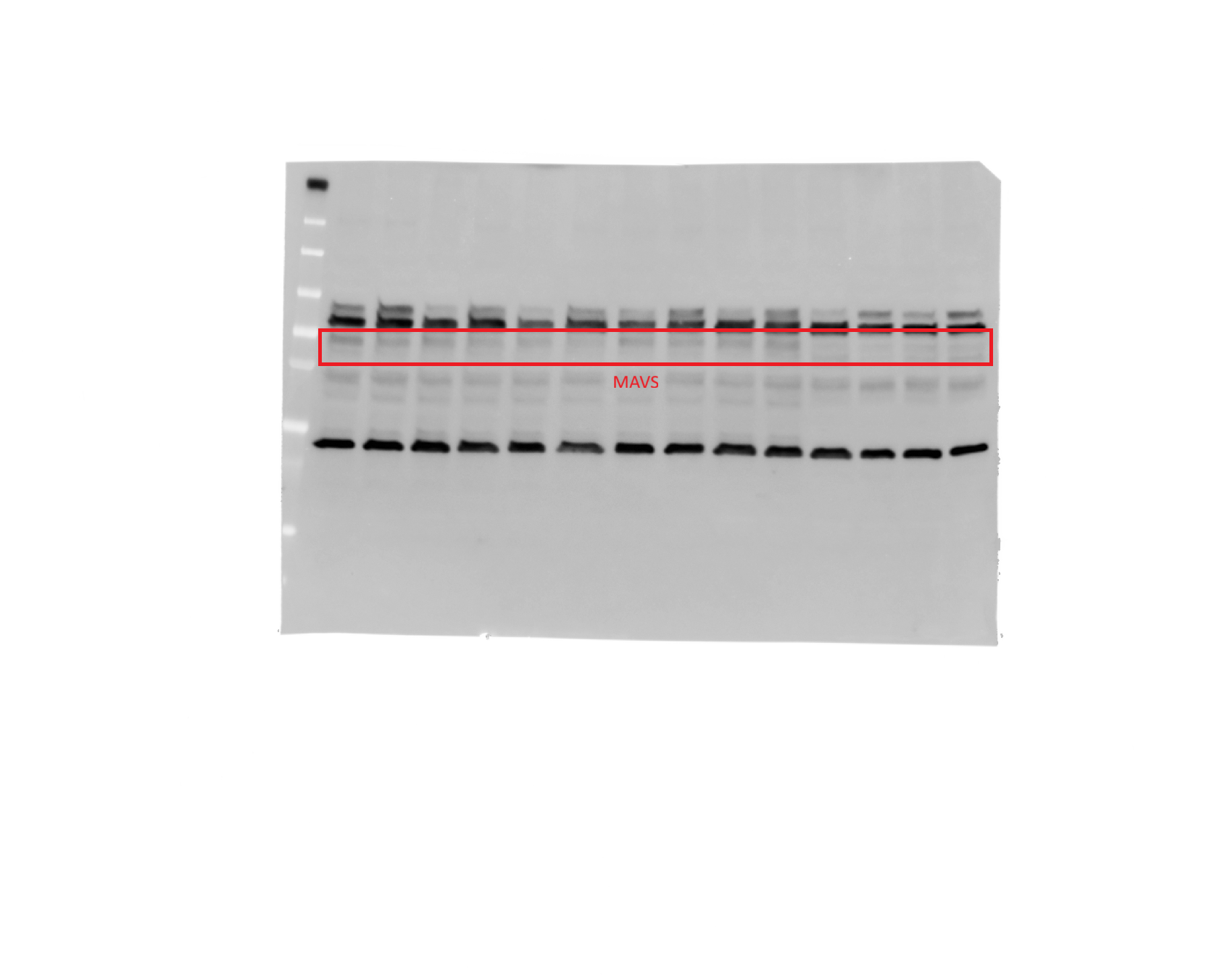

Supplement: Figure 2—figure supplement 1—source data 4. [file elife-106249-fig2-figsupp1-data4.zip › Figure 2-figure supplement 1-Source data 2_labelled blots/Figure 2-figure supplement 1c_MAVS_labelled.tif]

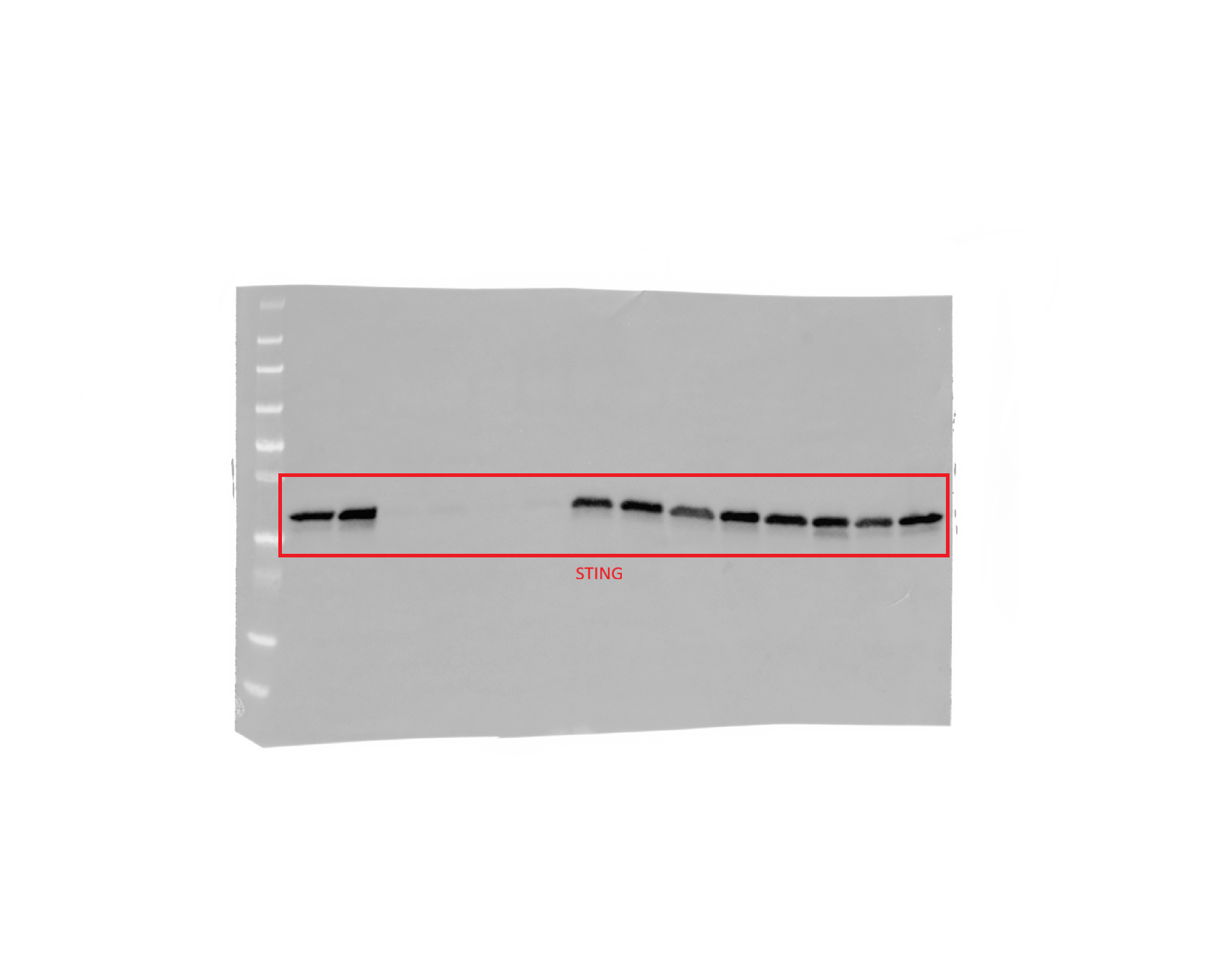

Supplement: Figure 2—figure supplement 1—source data 4. [file elife-106249-fig2-figsupp1-data4.zip › Figure 2-figure supplement 1-Source data 2_labelled blots/Figure 2-figure supplement 1c_STING_labelled.tif]

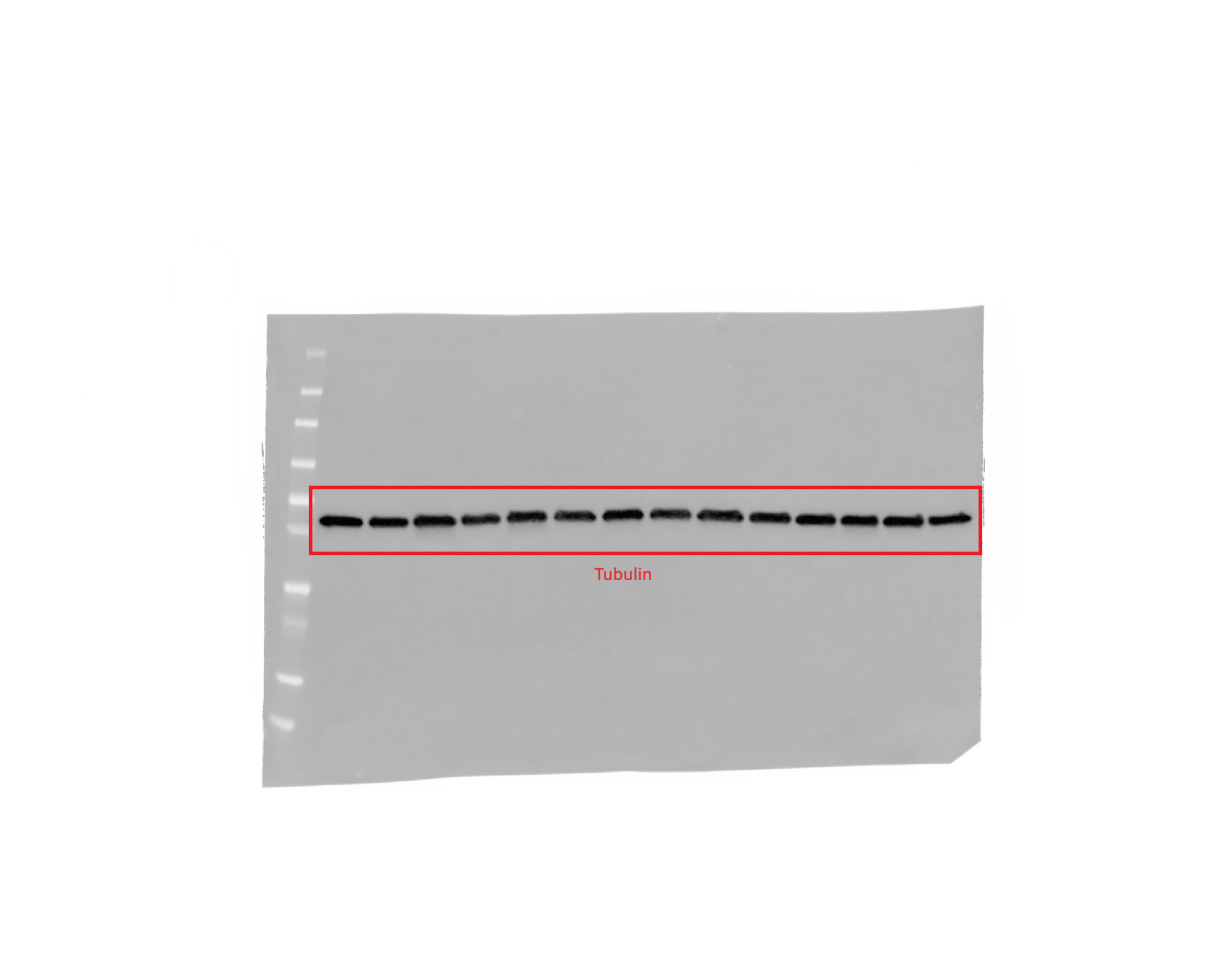

Supplement: Figure 2—figure supplement 1—source data 4. [file elife-106249-fig2-figsupp1-data4.zip › Figure 2-figure supplement 1-Source data 2_labelled blots/Figure 2-figure supplement 1c_Tubulin_labelled.tif]

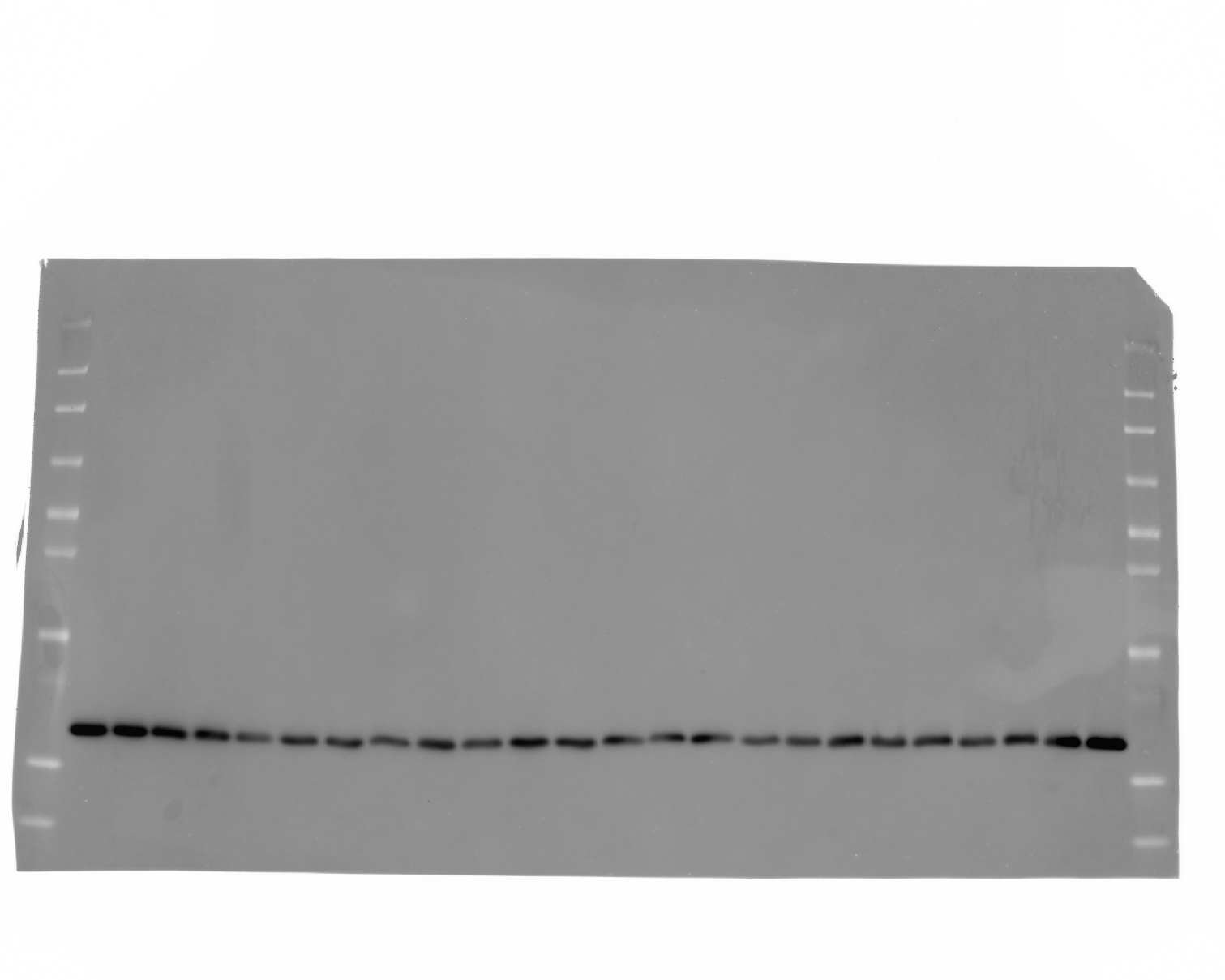

Supplement: Figure 2—figure supplement 1—source data 5. [file elife-106249-fig2-figsupp1-data5.zip › Figure 2-figure supplement 1-Source data 3_unlabelled blots/Figure 2-figure supplement 1i_H3_unlabelled.tif]

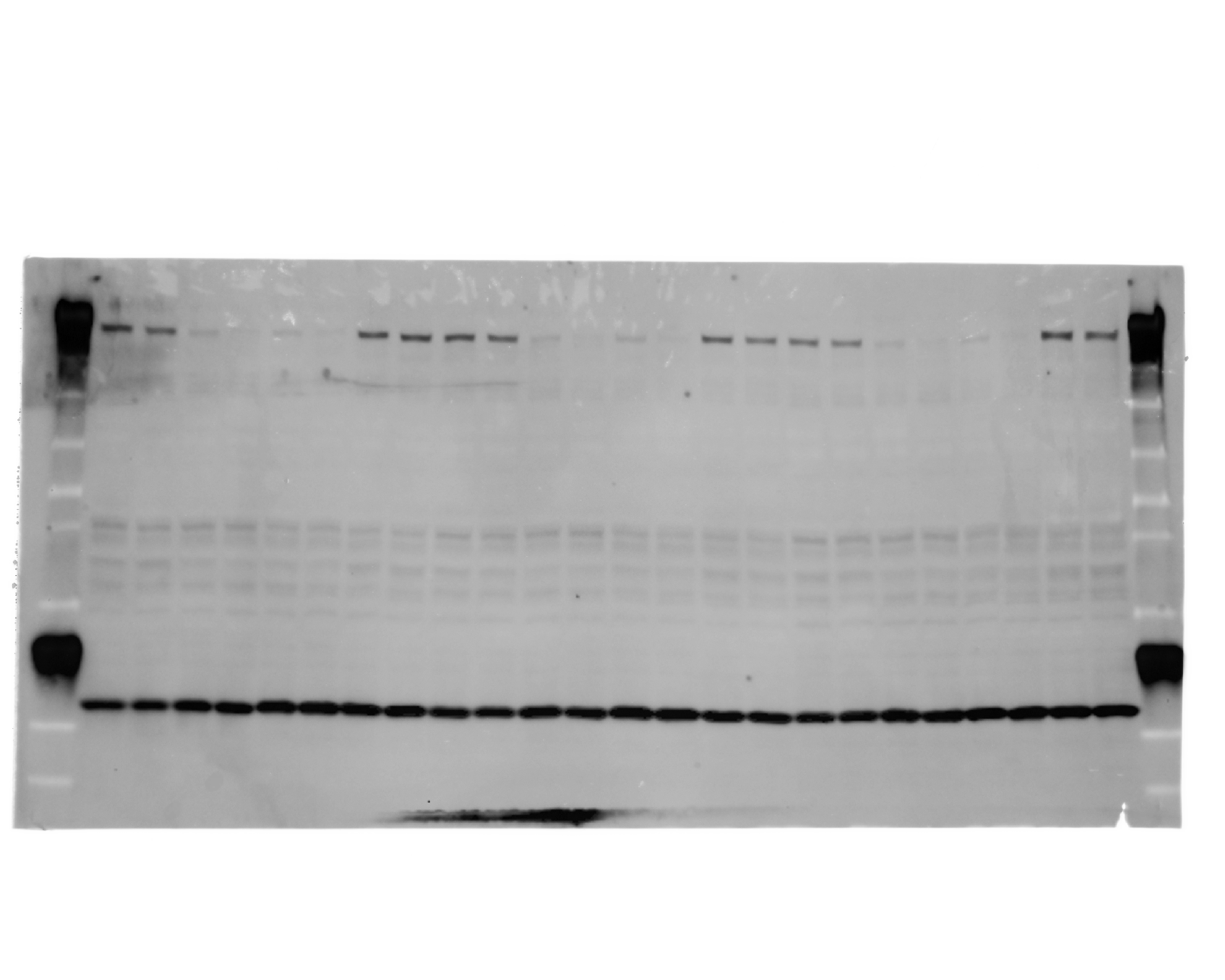

Supplement: Figure 2—figure supplement 1—source data 5. [file elife-106249-fig2-figsupp1-data5.zip › Figure 2-figure supplement 1-Source data 3_unlabelled blots/Figure 2-figure supplement 1i_KDM5A_unlabelled.tif]

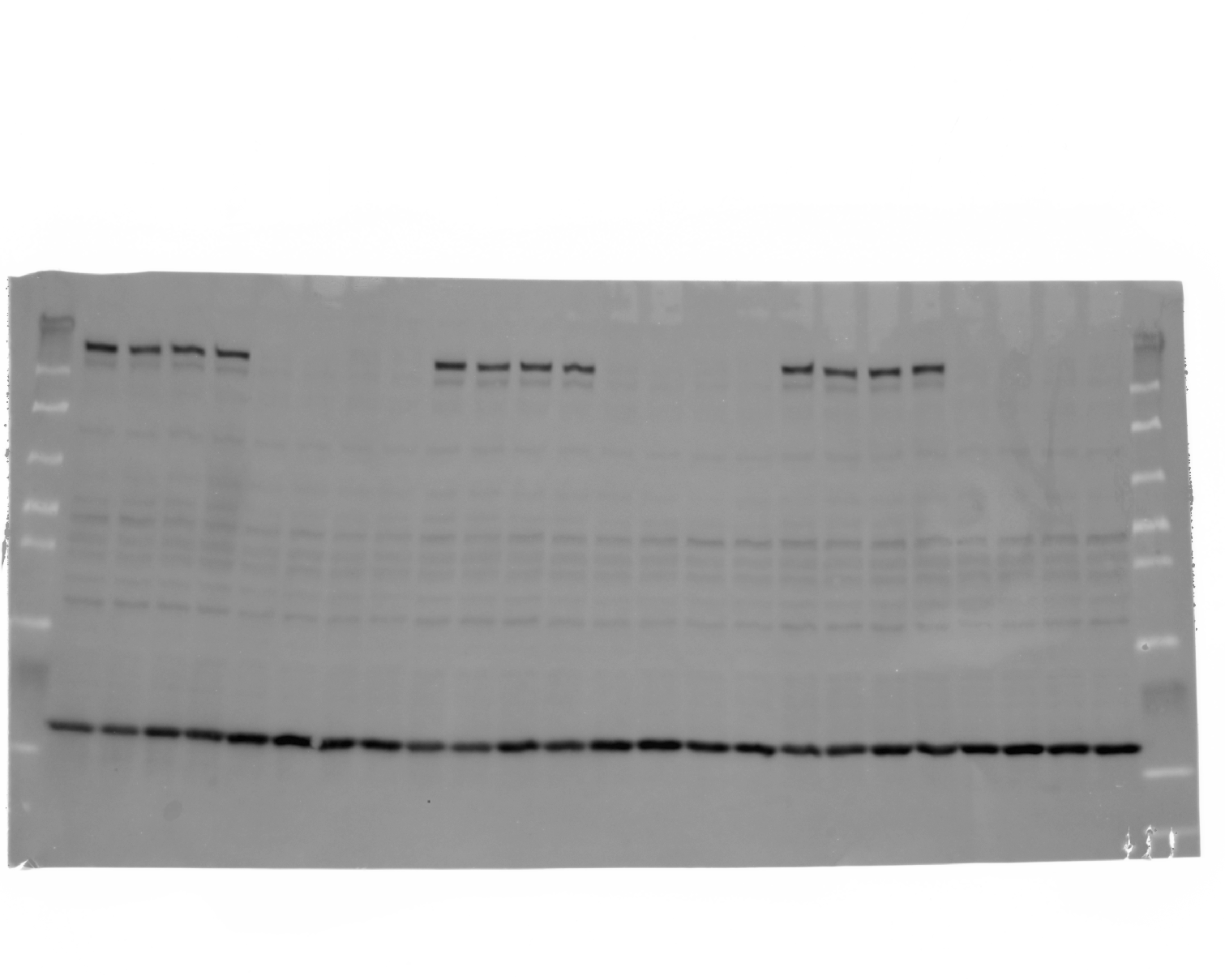

Supplement: Figure 2—figure supplement 1—source data 5. [file elife-106249-fig2-figsupp1-data5.zip › Figure 2-figure supplement 1-Source data 3_unlabelled blots/Figure 2-figure supplement 1i_KDM5C_unlabelled.tif]

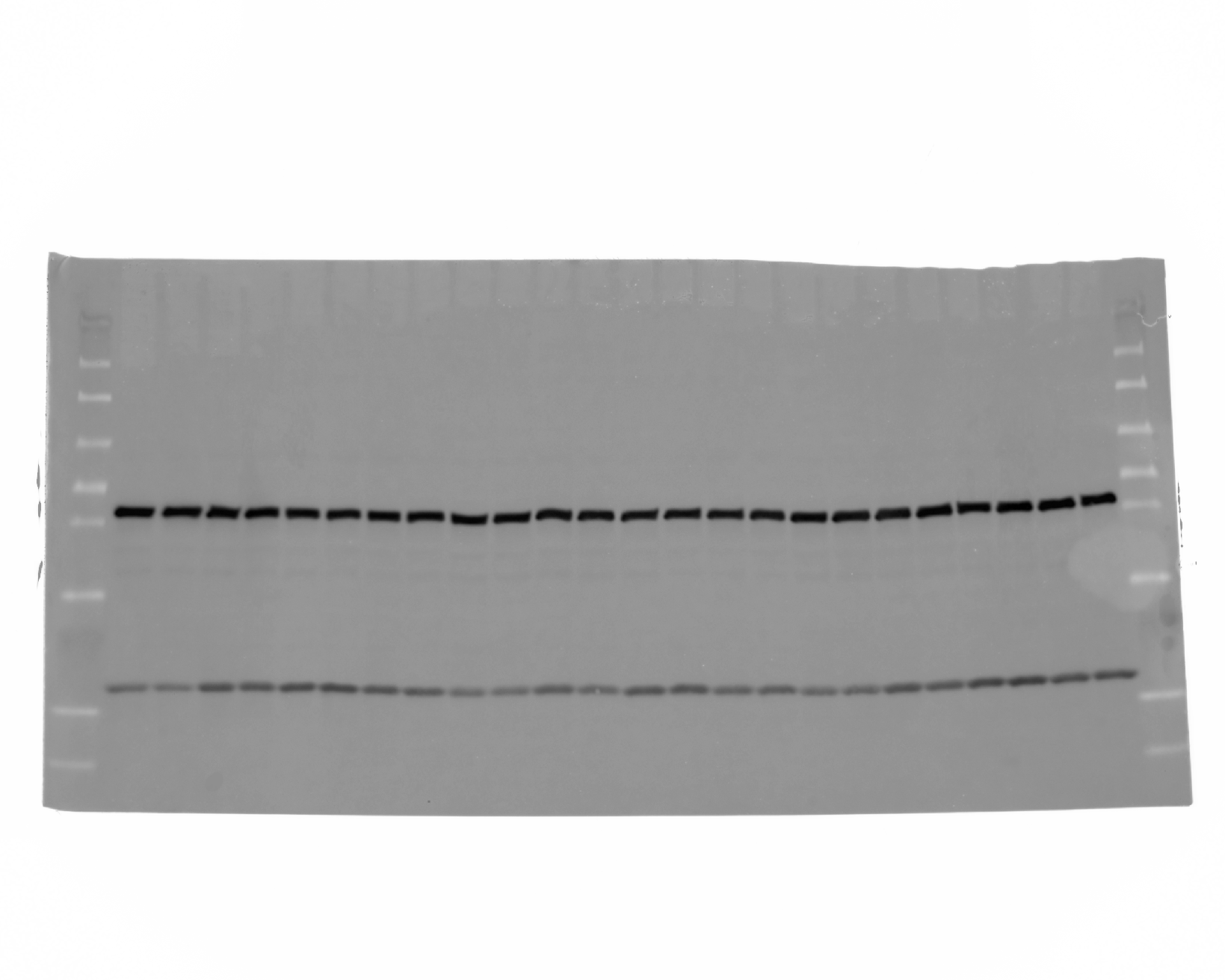

Supplement: Figure 2—figure supplement 1—source data 5. [file elife-106249-fig2-figsupp1-data5.zip › Figure 2-figure supplement 1-Source data 3_unlabelled blots/Figure 2-figure supplement 1i_Tubulin_unlabelled.tif]

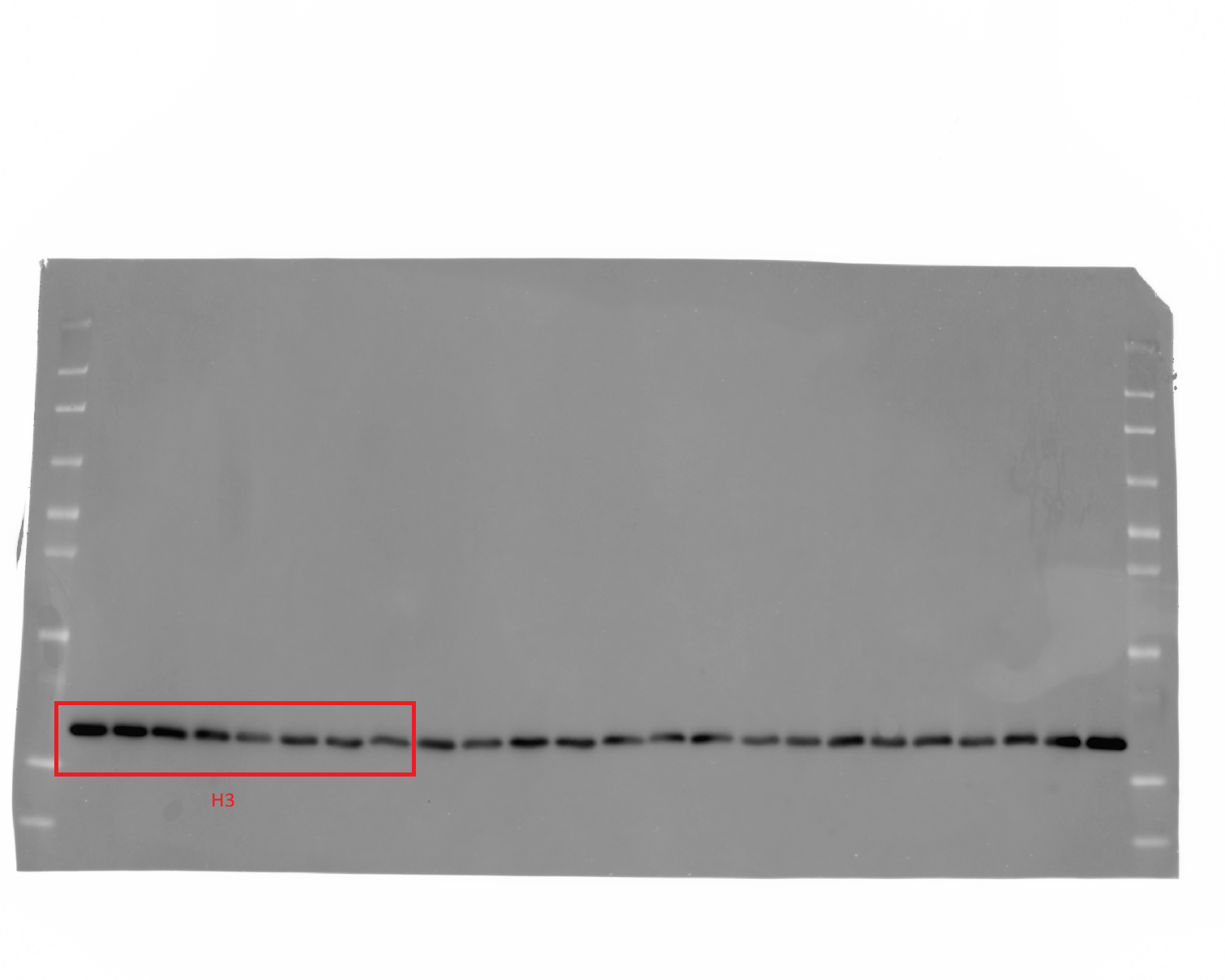

Supplement: Figure 2—figure supplement 1—source data 6. [file elife-106249-fig2-figsupp1-data6.zip › Figure 2-figure supplement 1-Source data 3_labelled blots/Figure 2-figure supplement 1i_H3_labelled.tif]

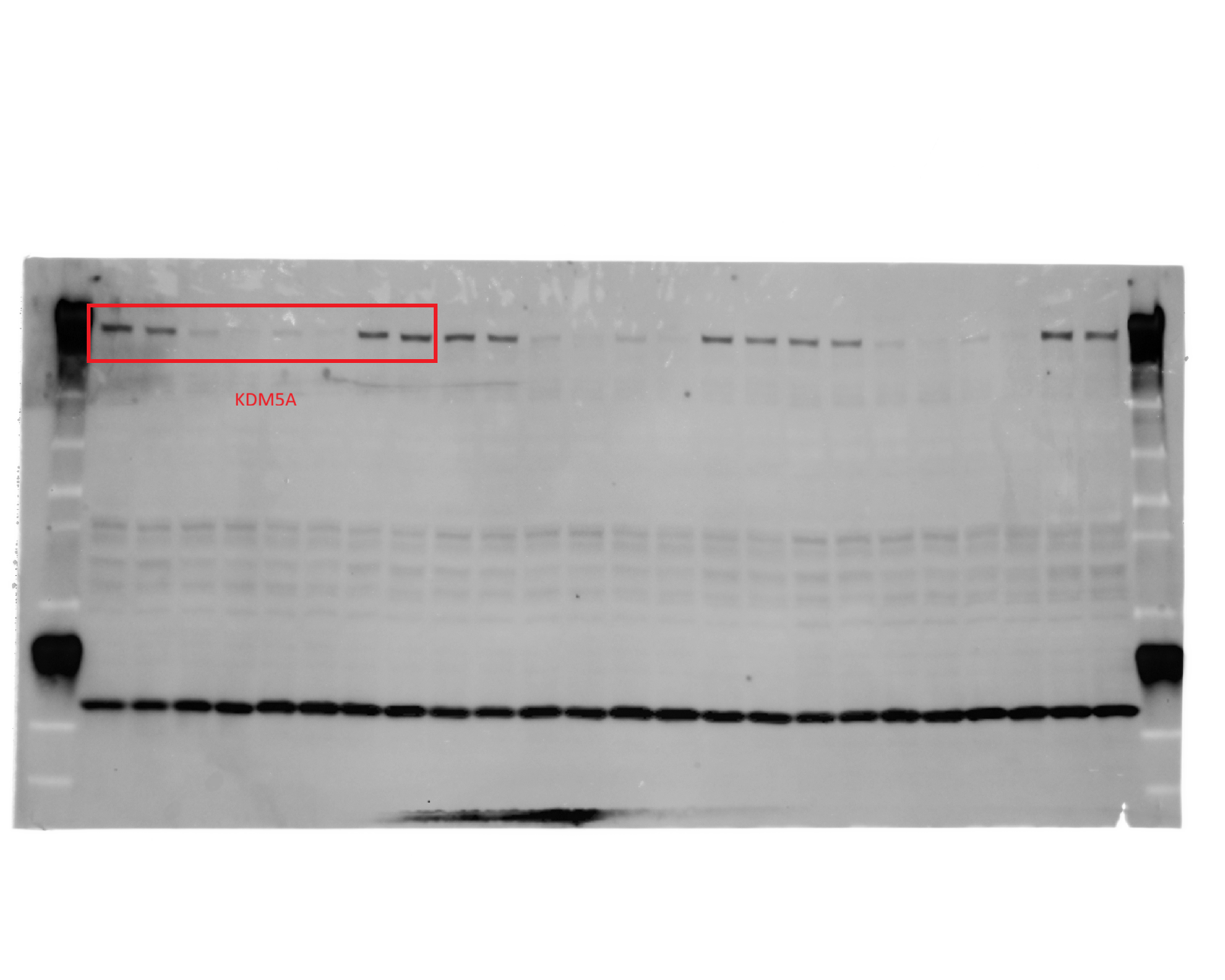

Supplement: Figure 2—figure supplement 1—source data 6. [file elife-106249-fig2-figsupp1-data6.zip › Figure 2-figure supplement 1-Source data 3_labelled blots/Figure 2-figure supplement 1i_KDM5A_labelled.tif]

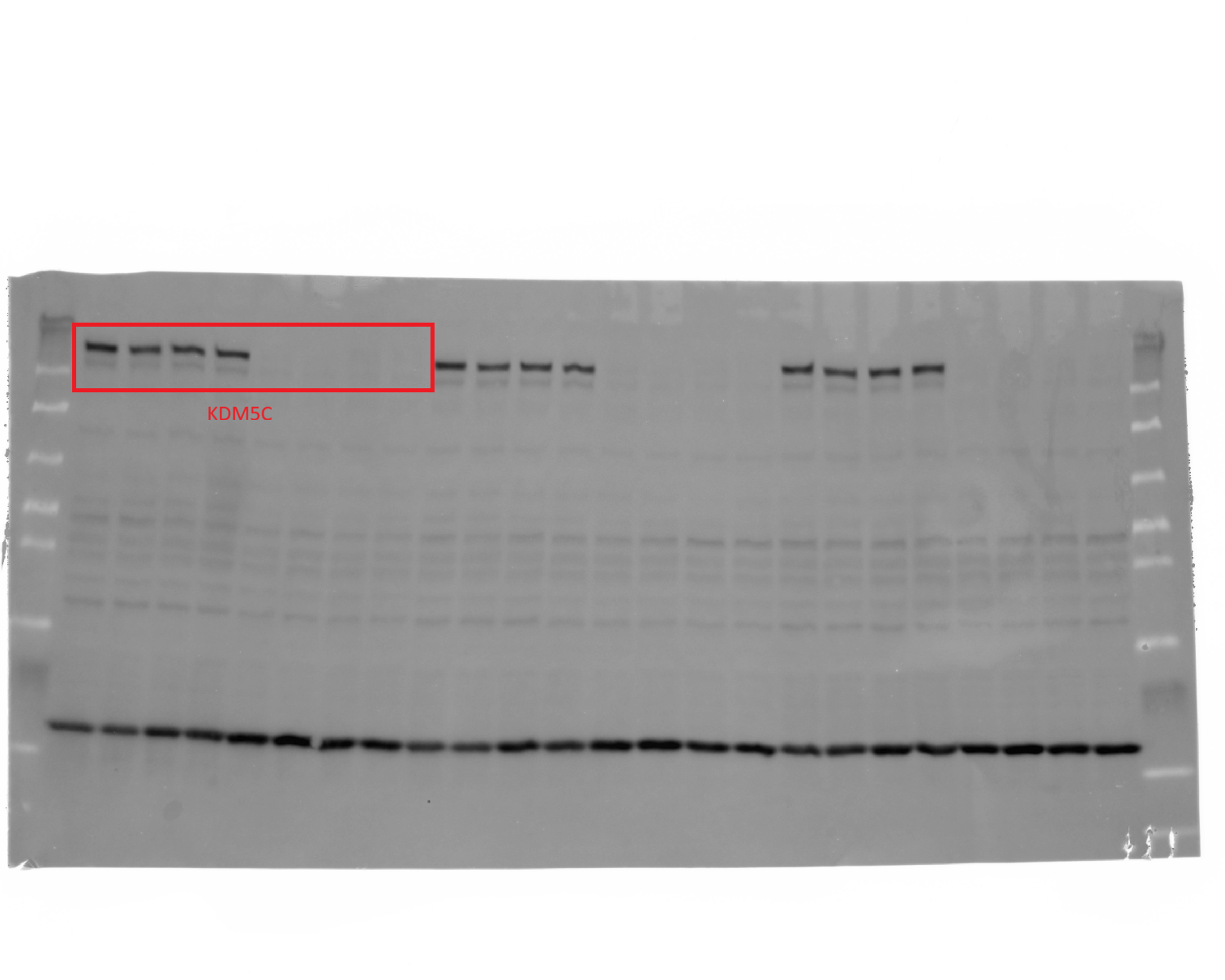

Supplement: Figure 2—figure supplement 1—source data 6. [file elife-106249-fig2-figsupp1-data6.zip › Figure 2-figure supplement 1-Source data 3_labelled blots/Figure 2-figure supplement 1i_KDM5C_labelled.tif]

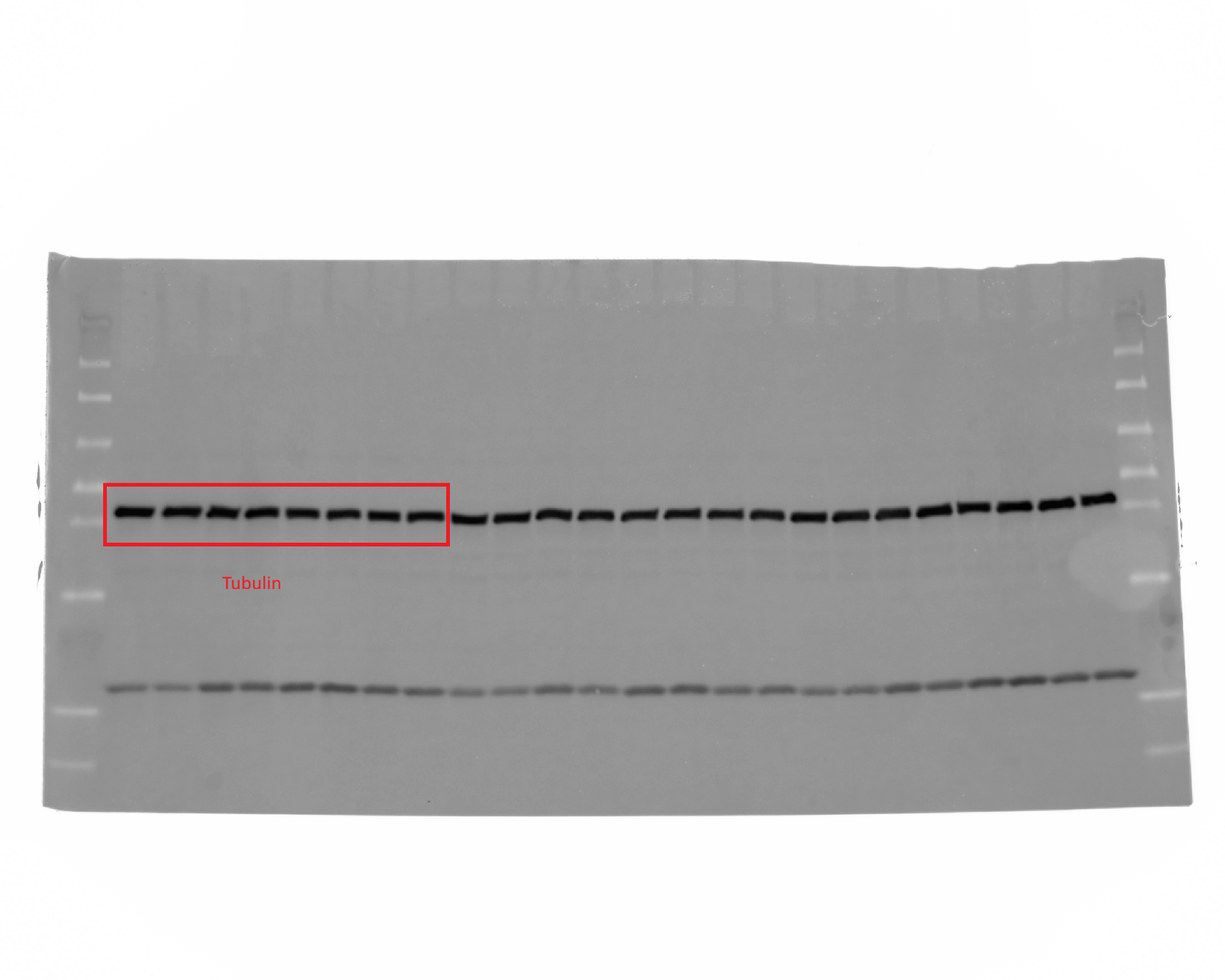

Supplement: Figure 2—figure supplement 1—source data 6. [file elife-106249-fig2-figsupp1-data6.zip › Figure 2-figure supplement 1-Source data 3_labelled blots/Figure 2-figure supplement 1i_Tubulin_labelled.tif]

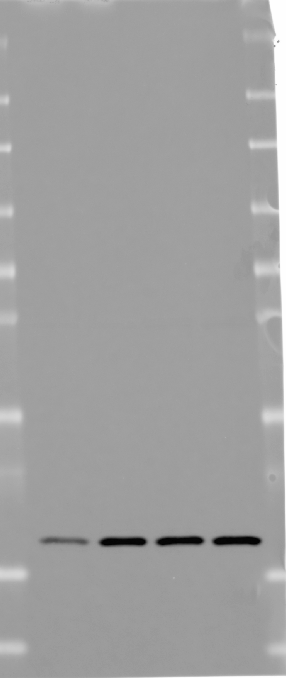

Supplement: Figure 3—source data 1. [file elife-106249-fig3-data1.zip › Figure 3-Source data 1_unlabelled blots/Figure-3a_H3K4me3_HCC1428_unlabelled.tif]

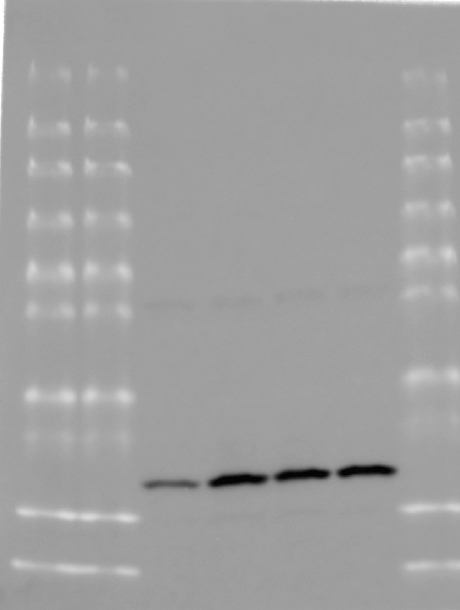

Supplement: Figure 3—source data 1. [file elife-106249-fig3-data1.zip › Figure 3-Source data 1_unlabelled blots/Figure-3a_H3K4me3_HMEC_unlabelled.tif]

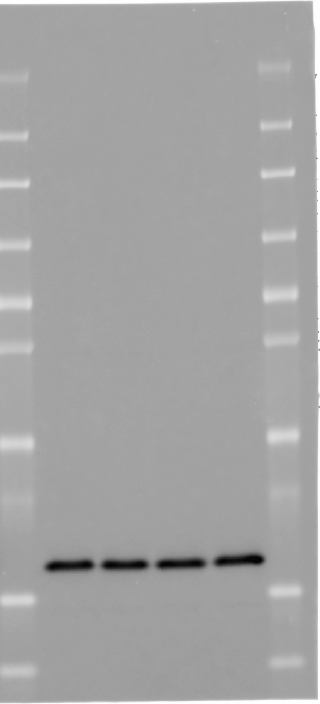

Supplement: Figure 3—source data 1. [file elife-106249-fig3-data1.zip › Figure 3-Source data 1_unlabelled blots/Figure-3a_H3_HCC1428_unlabelled.tif]

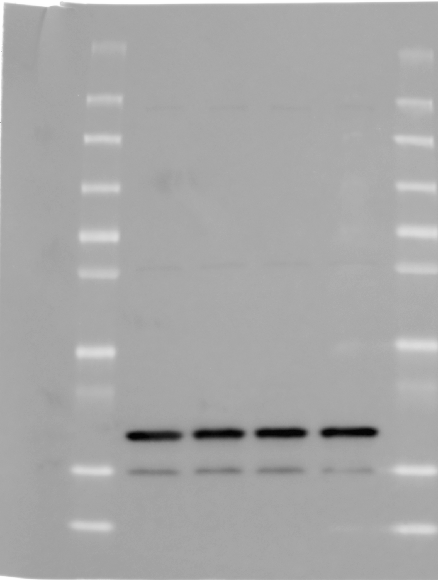

Supplement: Figure 3—source data 1. [file elife-106249-fig3-data1.zip › Figure 3-Source data 1_unlabelled blots/Figure-3a_H3_HMEC_unlabelled.tif]

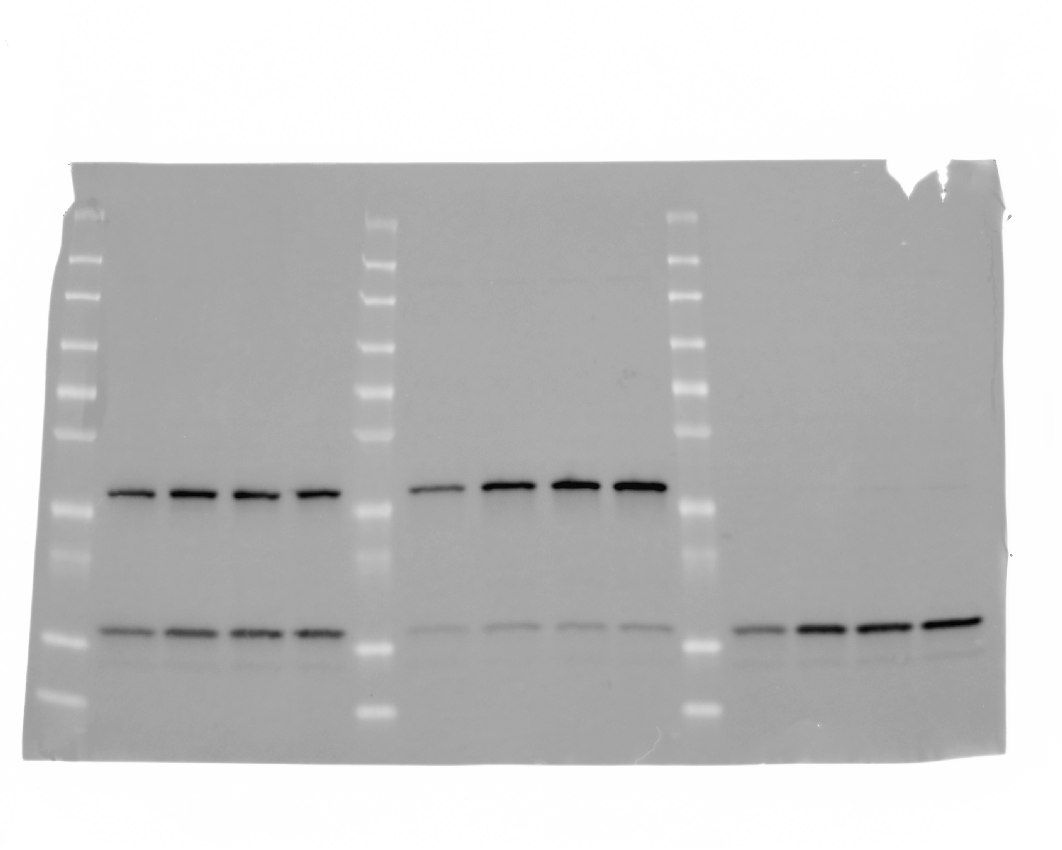

Supplement: Figure 3—source data 1. [file elife-106249-fig3-data1.zip › Figure 3-Source data 1_unlabelled blots/Figure-3a_STING_unlabelled.tif]

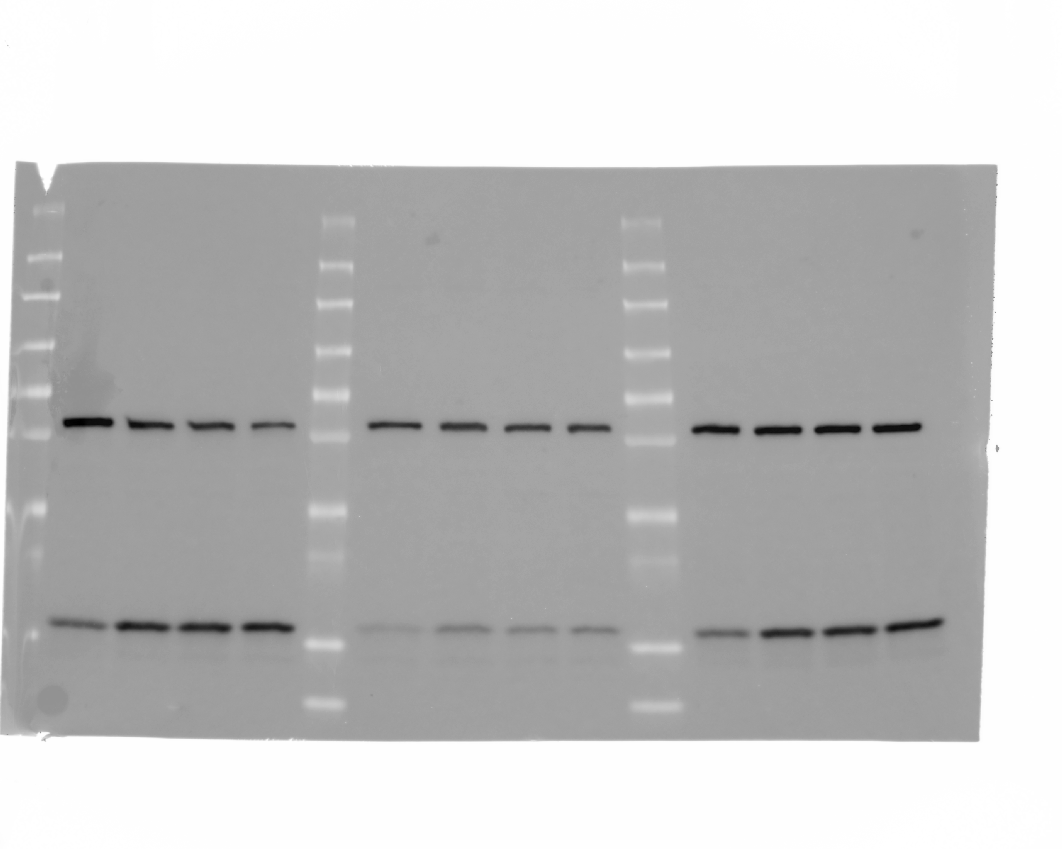

Supplement: Figure 3—source data 1. [file elife-106249-fig3-data1.zip › Figure 3-Source data 1_unlabelled blots/Figure-3a_tubulin_unlabelled.tif]

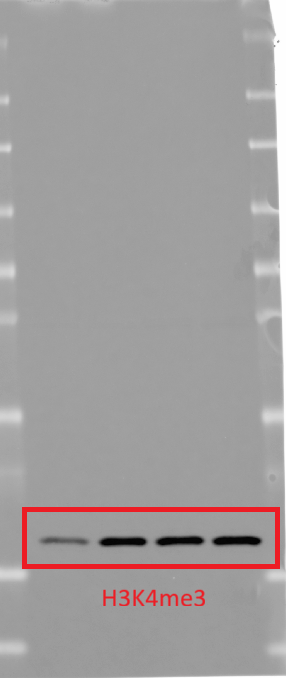

Supplement: Figure 3—source data 2. [file elife-106249-fig3-data2.zip › Figure 3-Source data 1_labelled blots/Figure-3a_H3K4me3_HCC1428_labelled.tif]

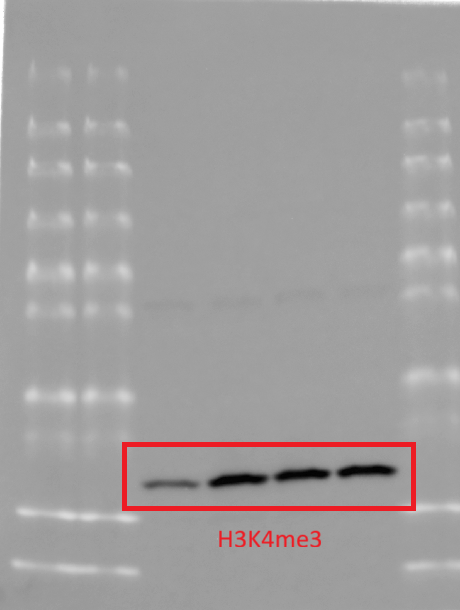

Supplement: Figure 3—source data 2. [file elife-106249-fig3-data2.zip › Figure 3-Source data 1_labelled blots/Figure-3a_H3K4me3_HMEC_labelled.tif]

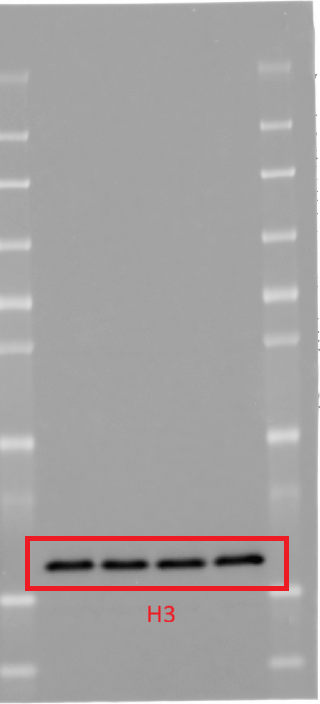

Supplement: Figure 3—source data 2. [file elife-106249-fig3-data2.zip › Figure 3-Source data 1_labelled blots/Figure-3a_H3_HCC1428_labelled.tif]

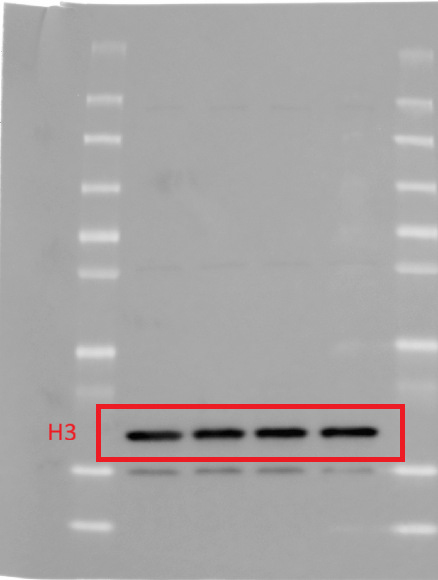

Supplement: Figure 3—source data 2. [file elife-106249-fig3-data2.zip › Figure 3-Source data 1_labelled blots/Figure-3a_H3_HMEC_labelled.tif]

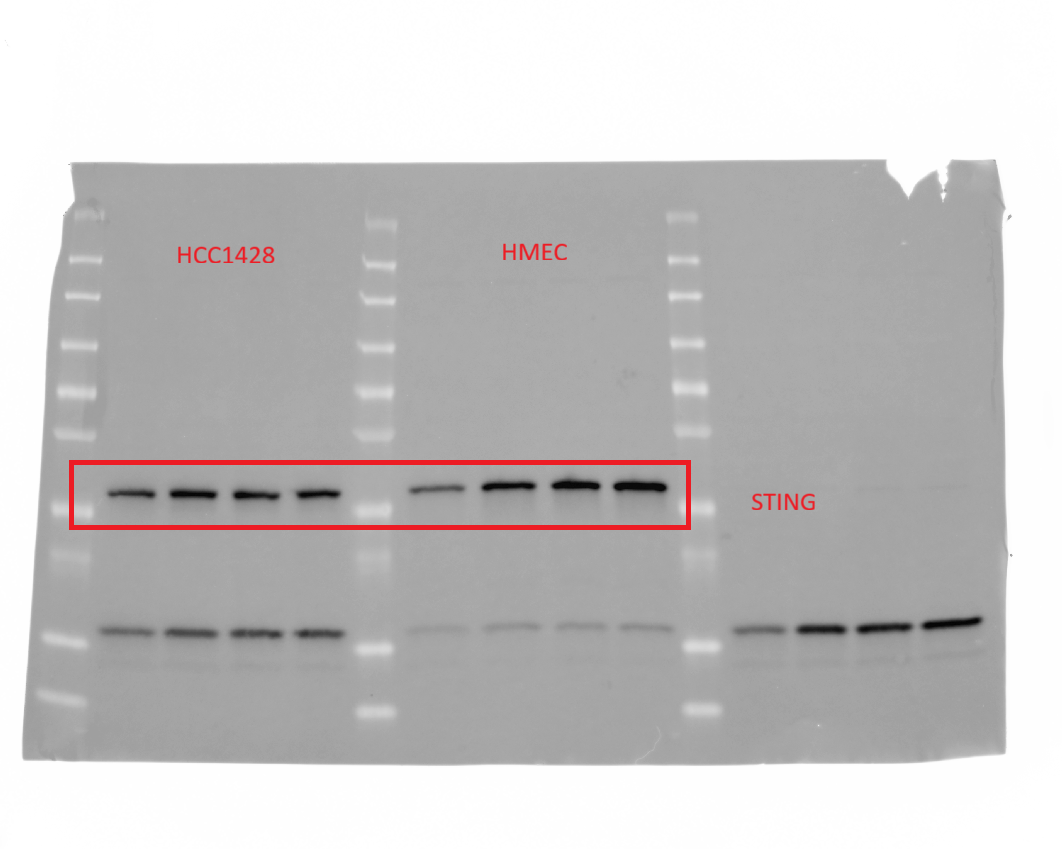

Supplement: Figure 3—source data 2. [file elife-106249-fig3-data2.zip › Figure 3-Source data 1_labelled blots/Figure-3a_STING_labelled.tif]

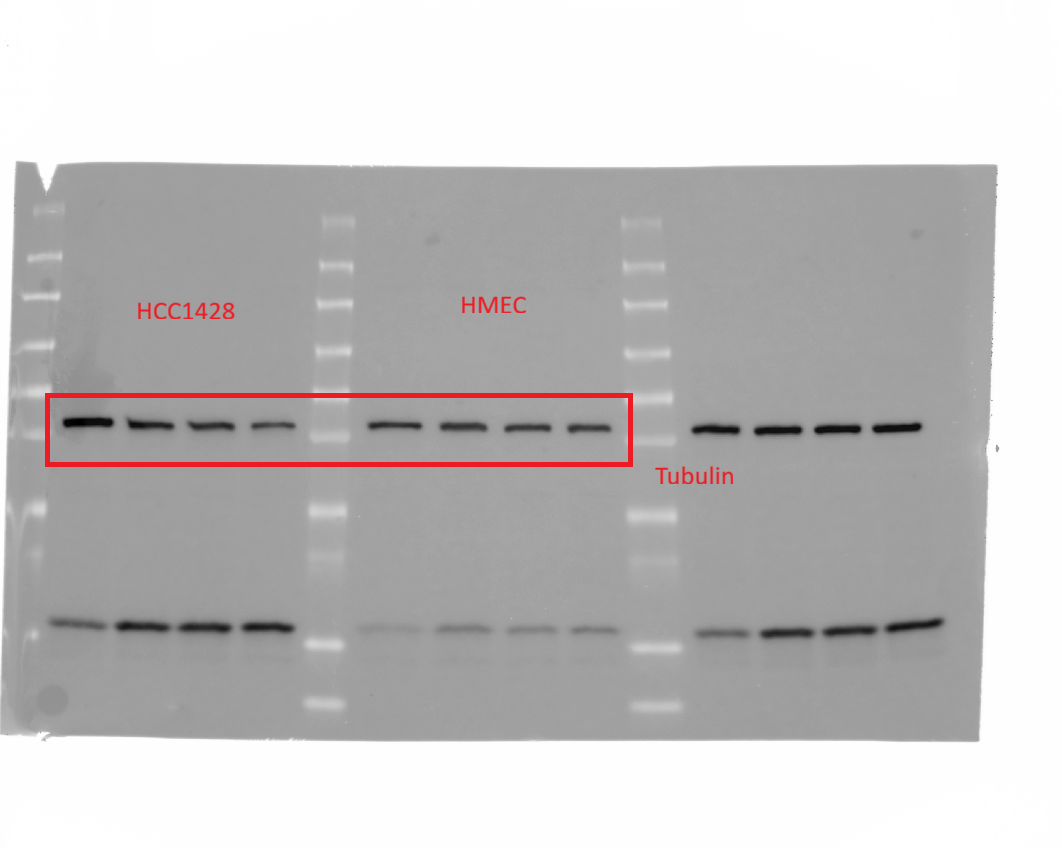

Supplement: Figure 3—source data 2. [file elife-106249-fig3-data2.zip › Figure 3-Source data 1_labelled blots/Figure-3a_tubulin_labelled.tif]

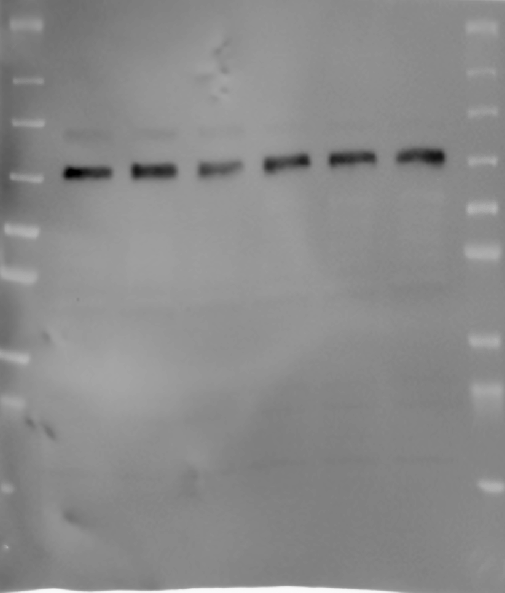

Supplement: Figure 4—source data 1. [file elife-106249-fig4-data1.zip › Figure 4-Source data 1_unlabelled blots/Figure 4c_Calnexin_unlabelled.tif]

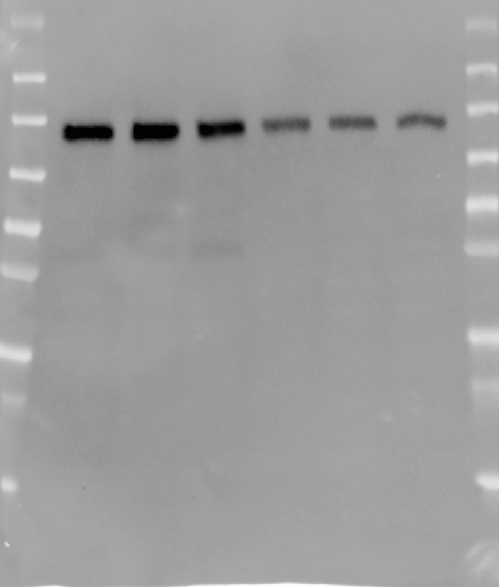

Supplement: Figure 4—source data 1. [file elife-106249-fig4-data1.zip › Figure 4-Source data 1_unlabelled blots/Figure 4c_XPF_unlabelled.tif]

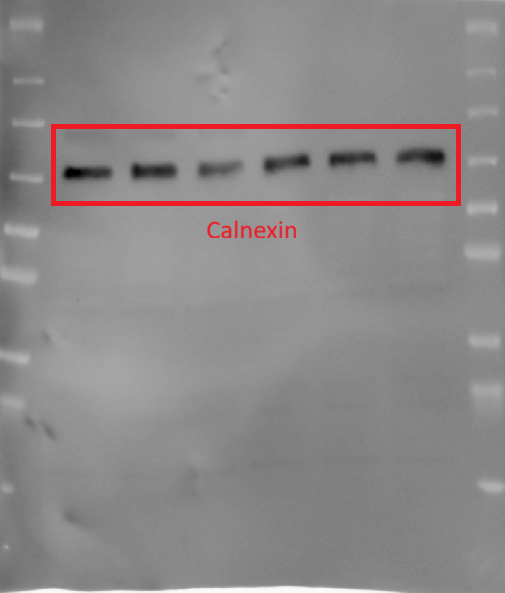

Supplement: Figure 4—source data 2. [file elife-106249-fig4-data2.zip › Figure 4-Source data 1_labelled blots/Figure 4c_Calnexin_labelled.tif]

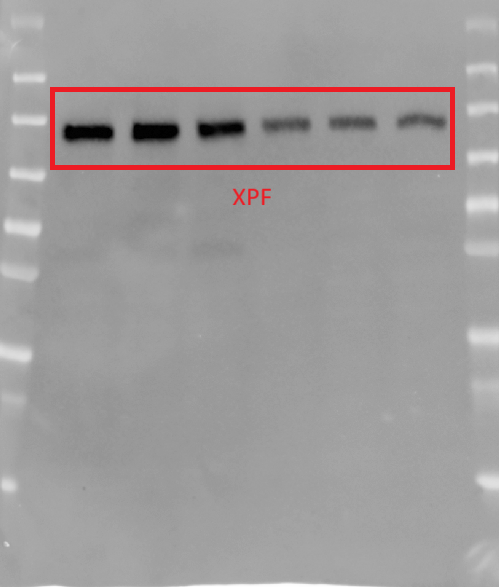

Supplement: Figure 4—source data 2. [file elife-106249-fig4-data2.zip › Figure 4-Source data 1_labelled blots/Figure 4c_XPF_labelled.tif]
